# Healing effect of curcumin on tooth extraction sockets in diabetic rats

**DOI:** 10.1590/1678-7757-2024-0251

**Published:** 2024-11-15

**Authors:** Tipthanan Chotipinit, Weera Supronsinchai, Soranun Chantarangsu, Supaporn Suttamanatwong

**Affiliations:** 1 Chulalongkorn University Faculty of Graduate School Bangkok Thailand Chulalongkorn University, Faculty of Graduate School, Interdisciplinary Program of Physiology, Bangkok, Thailand.; 2 Chulalongkorn University Faculty of Dentistry Department of Physiology Bangkok Thailand Chulalongkorn University, Faculty of Dentistry, Department of Physiology, Bangkok, Thailand.; 3 Chulalongkorn University Faculty of Dentistry Department of Oral Pathology Bangkok Thailand Chulalongkorn University, Faculty of Dentistry, Department of Oral Pathology, Bangkok, Thailand.

**Keywords:** Curcumin, Diabetes mellitus, Tooth extraction, Wound healing

## Abstract

Diabetes mellitus (DM) delays wound healing, including those following tooth extractions. Curcumin (CCM) can promote soft tissue and bone healing.

**Objective:**

The present study investigates the healing effects of CCM on tooth extraction sockets in diabetic rats.

**Methodology:**

Ninety-six male Wistar rats were divided into the following four groups: Control+Corn Oil (CO), Control+CCM, DM+CO, and DM+CCM. Each group was subdivided into 7-, 14-, and 28-day time point subgroups comprising eight rats. All animals had their maxillary first molars extracted. CCM-treated rats received 100 mg/kg of CCM orally for 7, 14, and 28 days. The lesion area was evaluated using macroscopic analyses, whereas socket healing was assessed by hematoxylin and eosin staining. Keratinocyte growth factor (KGF), Runt-related transcription factor 2 (Runx2), and collagen type I (COL1) expression levels were obtained using quantitative polymerase chain reaction (qPCR). Bone healing was analyzed by means of microcomputed tomography (μCT).

**Results:**

After 7 days, the groups showed no significant differences in lesion area and by day 14, no lesions were present. CCM treatment increased KGF mRNA expression in diabetic rats; however, diabetic rats showed delayed bone healing unrelated to CCM. CCM treatment resulted in increased Runx2 mRNA expression only in control rats, whereas COL1 mRNA expression remained unaffected by CCM.

**Conclusion:**

CCM shows potential as a soft tissue healing enhancer in diabetic rats and could serve as an additional treatment to promote soft tissue repair in diabetic individuals. Although CCM did not impact alveolar bone healing, it may enhance bone healing in other skeleton regions.

## Introduction

Diabetes mellitus (DM), a chronic metabolic disorder characterized by elevated blood glucose levels, often leads to various complications.^1^ Tooth extraction in diabetic patients frequently results in delayed socket healing.^2–4^ Diabetic rats exhibit lower levels of collagen fibers, reduced connective tissue, and diminished bone formation in extraction sockets.^2^ Additionally, diabetic rats have a 40% lower alveolar bone density compared with non-diabetic counterparts, indicative of reduced new bone formation.^3^ Diabetes is also associated with decreased bone trabecular percentage and impaired re-epithelialization.^4^ Runx2, a member of the Runx family of transcription factors, plays a crucial role in osteoblast differentiation.^5^ Runx2 activation facilitates the expression of alkaline phosphatase (ALP), COL1, osteopontin (OPN), bone sialoprotein (BSP), and osteocalcin (OCN).^5^ However, reduced levels of Runx2 have been observed at peri-implant sites in diabetic rats.^6^ KGF plays a vital role in wound healing by stimulating keratinocyte migration and proliferation.^7^ Studies in genetically diabetic db/db mice have shown decreased KGF mRNA expression at the wound site, potentially impacting re-epithelialization.^8^

Curcumin (CCM), a polyphenolic compound, possesses diverse biological functions, including antioxidant and anti-inflammatory properties.^9^ Research has shown that CCM significantly promotes skin wound healing in rats.^10^ Moreover, investigations into its effects on bone health have revealed promising outcomes.^6,11^ CCM significantly enhanced the expression of Runx2, ALP, and OCN in MC3T3-E1 cells.^11^ Additionally, it has shown potential in enhancing bone volume and bone-implant contact (BIC) in diabetic rats.^6^ However, the impact of CCM on the healing process in diabetic rats following tooth extraction remains unclear; therefore, this study investigated the effects of CCM on post-tooth extraction healing in diabetic rats at different time points.

## Methodology

### Study groups and experimental design

This study was approved by the Institutional Animal Care and Use Committee (IACUC). Sample size was calculated using the G Power analysis program to achieve 80% power (1-β) with a 95% confidence interval (α=0.05).^3^ A total of 96 male Wistar rats (8–12 weeks old, weight: 250–350 g) were randomly divided into four groups: Control+CO, Control+CCM, DM+CO, and DM+CCM, then further subdivided into 7-, 14-, and 28-day time points with eight rats per group. Rats were housed two per cage and acclimated for one week in a temperature- and humidity-controlled room with a 12-hour light/dark cycle before experimentation, with *ad libitum* access to food and water*.* Body weight and food intake were consistently monitored throughout the experiment. A total of six animals died after surgery and oral gavage.

### DM induction

DM was induced in rats via intraperitoneal injection of streptozotocin (STZ; Sigma, St Louis, MO, USA) administered at a dose of 65 mg/kg, dissolved in 0.09M citrate buffer (Sigma). Control rats received only the citrate buffer. Fasting blood glucose (FBG) levels were measured 48 hours after STZ injection (pre-operation) and at 7, 14, and 28 days after tooth extraction and CCM treatment (post-operation). FBG levels exceeding 200 mg/dL after a 6-hour fast indicated DM, measured at tail veins using an Accu-Chek Guide glucometer (Roche, Indianapolis, IN, USA). Rats with FBG levels below 200 mg/dL following STZ injections were excluded from the study.

### Tooth extraction and CCM treatment

After seven days of blood glucose monitoring, the animals were anesthetized with 40 mg/kg of zoletil (Virbac Laboratories, Carros, France) and 2 mg/kg of xylazine (Bic Chemical, Kamphaeng Saen, Nakhon Pathom, Thailand) via intraperitoneal injection. Their maxillary first molars were extracted using a dental explorer. Two hours later, CCM-treated rats received daily oral gavage of 100 mg/kg CCM dissolved in CO (Sime Darby Oils Morakot Public Company Limited, Klongtoey, Bangkok, Thailand), whereas control rats received CO for 7, 14, and 28 days. Tramadol (T.P. drug laboratories (1696), Phra Khanong, Bangkok, Thailand) was administered subcutaneously at a 4 mg/kg dosage for 5 days for pain relief. All experimental procedures were performed by a single investigator. Rats in which root fracture occurred during extraction were immediately excluded from the study.

### Lesion area analysis

Rats were euthanized via carbon dioxide inhalation. Maxillae were excised and rinsed with ice-cold phosphate-buffered saline. After photographing the lesion area, standardization and calculation were performed using Image J Software (National Institute of Health, Bethesda, MD, USA). Results were expressed as a percentage relative to the control group.

### μCT evaluation

After a microscopic study, the left maxilla was fixed in 4% paraformaldehyde (Merck, Darmstadt, Germany) at 4°C for 48 hours. For bone formation assessment, the maxilla underwent μCT scanning (μCT35 Scanco Medical AG, Brüttisellen, Switzerland) at 70 kV/100 μA with a slice thickness of 10 μm. Bone formation was assessed by outlining a region of interest (ROI) within a μCT image. The ROI was expanded mesially from the mesial surface of the second molar to the extraction site, incorporating the interradicular bone. μCT evaluation was performed to measure trabecular bone formation in 150 slices covering the whole extraction socket area. This evaluation determined bone volume/tissue volume (BV/TV), bone mineral density (BMD), trabecular thickness (Tb. Th, mm), trabecular number (Tb.N, mm), and trabecular separation (Tb. Sp, mm).

### Histomorphometry analysis

After μCT analysis, the left maxilla underwent decalcification in 10% ethylenediaminetetraacetic acid (Ajax Finechem, New South Wales, Australia) for 10 days at room temperature. Subsequently, it was processed and embedded in paraffin. Sections of 5 μm thickness were cut in a buccal-palatal direction using a microtome and stained with hematoxylin (C.V. Laboratories, Bangkok Noi, Bangkok, Thailand) and eosin (Bio-Optica S.p.A., Milan, Italy). Observation of the histological slides used an optical microscope (Carl Zeiss AG, Oberkochen, Baden-Württemberg, Germany). All samples were blinded during analysis by the investigator.

### RNA extraction and RT-qPCR

Right alveolar bone and gingival tissue RNA were extracted using Trizol reagent (Invitrogen, Waltham, MA, USA) per the manufacturer's instructions. Reverse transcription into cDNA utilized the RevertAid RT Reverse Transcription Kit (Thermo Scientific, Waltham, MA, USA). cDNA template underwent amplification by Luna Universal qPCR Master Mix (BioLab, Ipswich, MA, USA) with a 45-cycle PCR program including denaturation, annealing, and extension steps. Samples were analyzed in duplicate, employing the comparative cycle threshold (CT) method with β-actin mRNA as the internal control. Fold change was calculated using the 2−ΔΔCT method, using control sample CT values as reference. PCR primers used were: KGF (forward 5’-GTAGCGATCAACTCAAGG-3’ and reverse 5’-GTACCACTGGGTGCGA-3’), Runx2 (forward 5’-TCTTCACAAATCCTCCCC-3’ and reverse 5’-TGGATTAAAAGGACTTGGTG-3’), COL1 (forward 5’-CCTGGCTCTCCTGGCTCTC-3’ and reverse 5’-TCAATCACTGTCTTGCCCCA-3’) and β-actin (forward 5’-ACGGTCAGGTCATCACTATCG-3’ and reverse 5’-GGCATAGAGGTCTTTACGGATG-3’)

### Statistical analysis

Data were presented as mean ± standard deviation and assessed for normality using the Shapiro-Wilk test. Statistical analyses involved the three-way ANOVA followed by the Tukey-Kramer test for multiple comparisons. Threshold for statistical significance was set at *P*<0.05 using GraphPad Prism 8 Software (GraphPad Software, Inc., San Diego, CA, USA).

## Results

### CCM did not affect FBG levels

**Figure 1:**
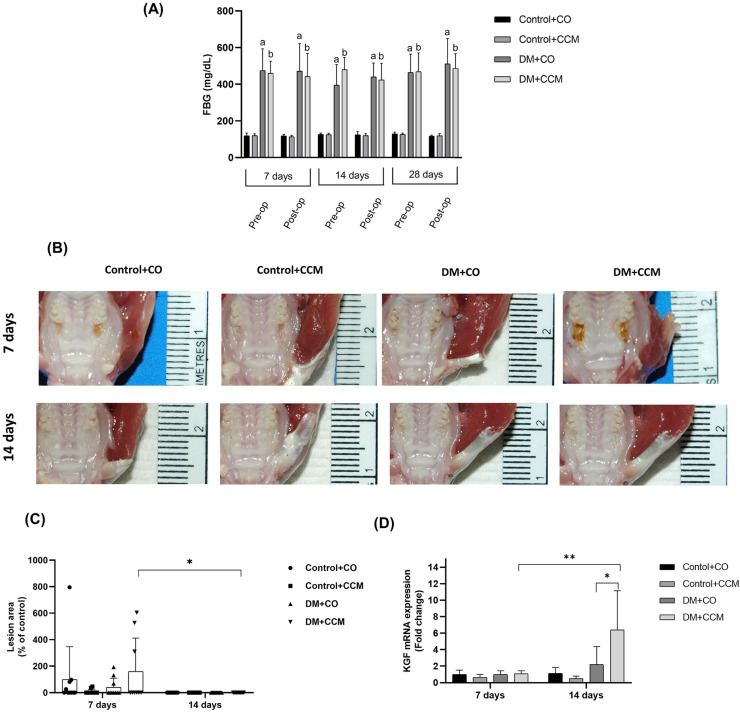
Effects of CCM treatment on FBG (A), soft tissue healing (B), lesion area (C), and KGF mRNA expression (D) in each group at 7, 14, and 28 days. Values are expressed as mean ± SD. ^a^P<0.0001 compared with Control+CO; ^b^P<0.0001 compared with Control+CCM; *P<0.05; **P<0.01; DM = diabetes mellitus; CCM = curcumin; CO = corn oil; FBG = fasting blood glucose; Pre-op = pre-operation; Post-op = post-operation; KGF = keratinocyte growth factor; SD = standard deviation; • = Control+CO; ■ = Control+CCM; ▲ = DM+CO; ▼= DM+CCM.

Following STZ injection, FBG levels significantly increased in the DM+CO and DM+CCM groups compared with the Control+CO and Control+CCM groups, respectively (*P*<0.0001). After 7, 14, and 28 days of CCM treatment, FBG levels remained elevated in the DM+CCM group compared with the Control+CCM group (*P*<0.0001) without any significant difference found between the DM+CCM and DM+CO groups (Figure 1A).

### Effects of CCM on soft tissue healing

Lesion areas did not significantly differ between groups at 7 days. All groups showed complete lesion healing by 14 days. However, only the DM+CCM group exhibited significantly different lesion areas at 14 days compared with 7 days (*P*=0.028), suggesting faster soft tissue healing between day 7 and day 14 (Figures 1B and 1C).

### CCM upregulated KGF mRNA expression in diabetic rats

KGF mRNA expression did not differ significantly among groups 7 days after tooth extraction. At 14 days, no significant difference was observed between Control+CCM and Control+CO, nor between DM+CO and Control+CO. However, KGF mRNA in DM+CCM was significantly upregulated compared with DM+CO (*P*=0.012). Moreover, KGF mRNA expression in the DM+CCM group at 14 days significantly increased compared with levels at 7 days (*P*=0.004; Figure 1D).

### Histomorphometric analysis of the sockets following CCM treatment

**Figure 2:**
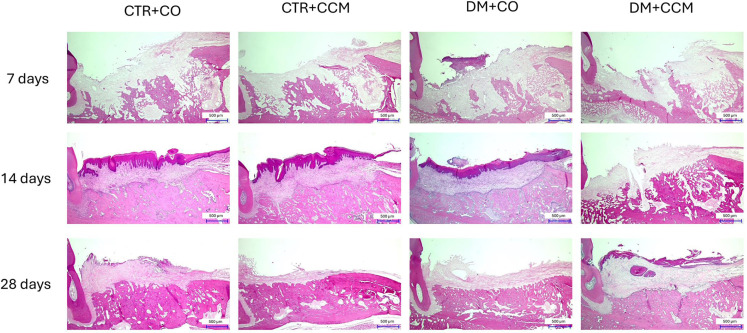
Effects of CCM treatment on histomorphometry analysis of the left tooth extraction sockets in each group at 7, 14, and 28 days with a scale bar of 500 μm. DM = diabetes mellitus; CCM = curcumin; CO = corn oil.

At 7 days, Control+CO and Control+CCM showed reduced levels of inflammation compared with diabetic rats. DM+CO had heightened inflammation, whereas those treated with CCM exhibited mitigated inflammatory responses. Collagen deposition was observed across all experimental groups, with the greatest accumulation seen in the Control+CCM group. Hemorrhage was evident in the Control+CO group, whereas vascular congestion occurred only in diabetic rats. Woven bone formation was consistent across all experimental groups (Figure 2). At 14 days post-extraction, all experimental groups showed diminished infiltration of inflammatory cells concomitant with collagen deposition above the sockets. Moreover, newly developed blood vessels were present. Enhanced trabecular bone formation was evident in non-diabetic rats, with osteoblasts and osteoclasts lining the sockets. Reduced trabecular bone formation was observed at the apical sockets in the diabetic groups (Figure 2). At 28 days, there was no inflammation or collagen deposition above the sockets. Notably, the presence of newly formed blood vessels, mature bone containing osteocytes, and alveolar ridge remodeling was evident (Figure 2).

### CCM effects on bone healing at the sockets from μCT analysis

**Figure 3:**
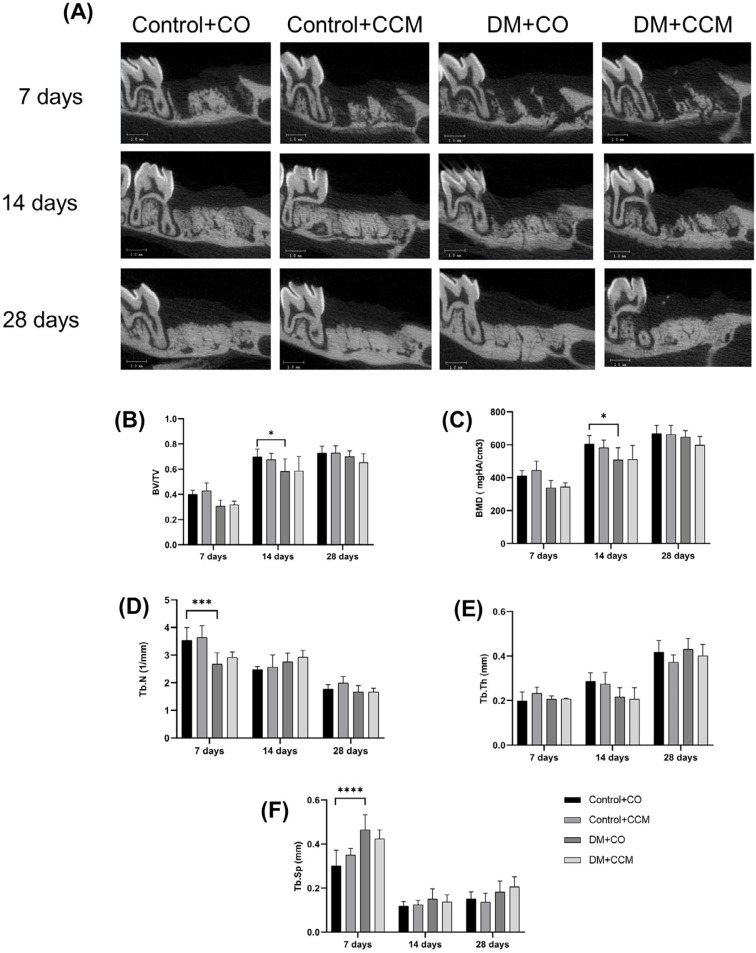
μCT images of each group at 7, 14, and 28 days with a scale bar of 1 mm (A). Effects of CCM treatment on BV/TV (B), BMD (C), Tb.N (D), Tb. Th (E), and Tb. Sp (F). Values are expressed as mean ± SD. *P<0.05; ***P<0.001; ****P<0.0001; μCT = microcomputed tomography; DM = diabetes mellitus; CCM = curcumin; CO = corn oil; BV/TV = bone volume per tissue volume; BMD = bone mineral density; Tb.N = trabecular number; Tb. Th = trabecular thickness; Tb. Sp = trabecular separation; SD = standard deviation.

Figure 3A presents μCT images post-tooth extraction in each group at three time points. At 7 days, Tb.N were significantly lower (*P*=0.006; Figure 3D) whereas Tb. Sp was significantly higher in the DM+CO group compared with the Control+CO group (*P*<0.0001; Figure 3F). No significant differences were observed among groups for BV/TV, BMD, and Tb. Th (Figures 3B, 3C, and 3E). At 14 days, only BV/TV (*P*=0.045; Figure 3B) and BMD (*P*=0.036; Figure 3C) were significantly lower in DM+CO compared with the Control+CO group, indicating delayed bone healing. At 28 days, all bone parameters from μCT did not differ significantly among groups (Figures 3B-F).

### CCM selectively increased Runx2 mRNA expression in tooth extraction sockets in non-diabetic rats

**Figure 4:**
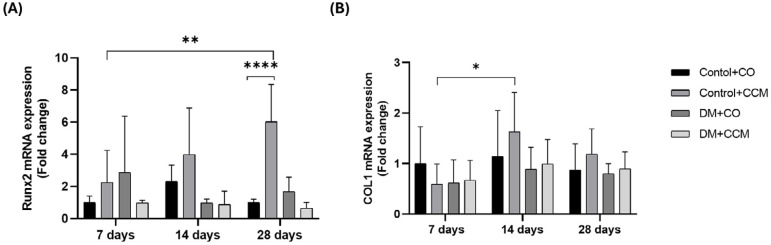
Effects of CCM treatment on Runx2 (A) and COL1 mRNA expression (B) in each group at 7, 14, and 28 days after tooth extraction. Values are expressed as mean ± SD. *P<0.05; **P<0.01; ****P<0.0001; DM = diabetes mellitus; CCM = curcumin; CO = corn oil; Runx2 = Runt-related transcription factor 2; COL1 = collagen type I; SD = standard deviation.

Runx2 mRNA expression did not differ significantly between all groups between 7 and 14 days, but it increased only in Control+CCM compared with Control+CO at 28 days (*P*=0.0001) and showed a significant increase from 7 to 28 days (*P*=0.007; Figure 4A). COL1 mRNA expression did not differ between all groups between 7 and 14 days. Interestingly, COL1 mRNA expression in the Control+CCM group at 14 days was significantly upregulated compared with Control+CCM at 7 days (*P*=0.016; Figure 4B).

## Discussion

Hyperglycemia causes vascular damage and impairs adequate blood perfusion.^12^ In the present study, CCM treatment did not reduce FBG levels. Prior studies showed conflicting findings regarding its effect on blood glucose levels in diabetic rats.^6,13^ Hence, its role in tooth extraction socket healing might not be linked to its glucose-regulating abilities in diabetes. Our study found larger lesion areas in diabetic rats treated with CCM at 7 days, but by day 14 all groups had healed completely which suggests that CCM may improve soft tissue healing after 7 days. Gene expression investigation revealed that CCM upregulated KGF mRNA expression in diabetic rats from 7 to 14 days which correlated with an increasing soft tissue healing rate. Thus, KGF may play a role in soft tissue healing after CCM treatment. Results showed that CCM selectively increased KGF mRNA expression in diabetic rats after tooth extraction. These results imply variable effects of CCM depending on the prevailing condition, as it has been previously reported that CCM protected against testicular damage exclusively in diabetic rats and inhibited inflammation only under high glucose conditions in retinal pigment epithelial cells.^14,15^

Alveolar bone healing represents an integral aspect of the overall process of tooth extraction wound healing.^16^ Our findings revealed time-dependent improvement in alveolar bone healing. At 14 days, however, diabetic rats showed a notable decrease in BV/TV and BMD, suggesting a delay in bone healing. Consistent with previous research, diabetes hinders alveolar bone repair, diminishing new bone formation and osteoblast numbers at the extraction site.^4^ Our study examined Runx2 and COL1 expression during alveolar bone healing and found that the expression patterns of these genes showed a correlation with bone formation within the sockets at 7 and 28 days, but not at 14 days. Our experiment revealed Runx2 mRNA upregulation in control rats treated with CCM for 28 days. Despite the observed increase in Runx2 expression, bone filling at the sockets was similar to that of control rats. This finding is consistent with a prior study indicating that CCM had no beneficial impact on fracture healing.^17^ Diabetic conditions did not affect Runx2 and COL1 mRNA expression at 7, 14, and 28 days post-extraction.

CCM did not exert a significant influence on alveolar bone healing in diabetic rats. However, previous research has shown that CCM treatment effectively reduces calvarial bone defects and enhances BIC and BV/TV at tibial peri-implant sites in diabetic rats.^6^ Presently, the data available on the mechanism of action of CCM in alveolar bone healing under diabetic conditions is limited, with prior investigations primarily focusing on bone resorption in periodontitis models.^18^ The alveolar bone serves as a vital supportive tissue and undergoes rapid remodeling in response to various stimuli such as mechanical force.^19^ Healing processes in both alveolar and calvarial bones primarily depend on intramembranous ossification with no involvement of cartilage formation.^20,21^ Calvarial bone regeneration utilizes osteoprogenitor-rich dura mater in conjunction with cells from the bone marrow, periosteum, and adjacent soft tissues, collectively contributing to enhance fracture repair.^22^ Femoral fracture healing depends on endochondral ossification, whereas alveolar bone lacks muscle stem cells crucial for long-bone fracture repair, indicating inherent differences in the healing mechanisms.^19,20^ In the present study, CCM showed no discernible impact on alveolar bone healing following tooth extraction. These findings suggest that CCM may have varying effects on bone healing processes and on the bone microenvironment across different skeletal sites, such as alveolar bone, calvaria, and long bones.

Our results show that CCM attenuates the exaggerated acute inflammatory response observed in diabetic rats following tooth extraction. Histological analysis revealed a reduction in inflammatory cell infiltration within the extraction site at day 7 post-extraction in the CCM-treated diabetic group compared with the diabetic control group. However, no significant difference in inflammatory cell infiltration was observed among any experimental group at the 14-day time point. Previous studies have consistently reported CCM effectiveness in mitigating inflammation during various stages, including both acute and chronic inflammation. CCM oral administration was found to reduce inflammation within the first six hours following experimentally induced zymosan-induced arthritis.^23^ CCM significantly suppressed bleomycin-induced acute lung injury by inhibiting inflammation.^24^ Moreover, treatment with 100 and 200 mg/kg of CCM for five days decreased immune cell infiltration and pro-inflammatory cytokines in acute lung injury.^25^ CCM has also been used as a therapeutic intervention in chronic inflammatory disorders such as rheumatoid arthritis.^25^ A daily dose of 1000 mg CCM for 8-12 weeks can alleviate chronic inflammation in arthritis models.^26^ Our finding suggests that CCM can potentially modulate the acute inflammatory process although further investigation is needed to elucidate the underlying mechanisms. Prior studies have indicated that CCM possesses an anti-inflammatory property involving pro-inflammatory cytokine suppression and chemokine production.^27,28^ Several CCM anti-inflammatory mechanisms have been reported including reducing monocyte chemoattractant protein-1 (MCP-1), a chemokine implicated in the recruitment of inflammatory cells, inhibition of cyclooxygenase-2 (COX-2), lipoxygenase (LOX), and inducible nitric oxide synthase (iNOS), key enzymes involved in inflammatory processes.^27,29^ Additionally, CCM may mitigate the inflammatory response by reducing chemokine production via NF-κB, MAPK, AP-1, JAK/STAT and other signaling pathways, thereby limiting immune cell infiltration.^27,30^ Moreover, the antioxidant properties of CCM are beneficial, as oxidative stress is a known contributor to inflammation.^28^

Previous research has classified CCM as safe.^26,31^ Systematic studies funded by the Prevention Division of the US National Cancer Institute (NCI) found that oral doses of CCM up to 3,500 mg/kg/day for up to 90 days produced no adverse effects in rats, dogs, or monkeys.^32^ Clinical trials indicate that to achieve maximum therapeutic effects CCM dosages should range between 4,000 and 8,000 mg per day.^26^ In human studies, CCM administered at 8 grams per day orally for three months was not toxic.^33^ Additionally, research has shown that oral administration of 100 mg/kg CCM for 30 days increased bone volume at the peri-implant site of the tibia and reduced alveolar bone loss due to experimental periodontitis in rats, all without causing toxic effects.^6,34,35^ Numerous trials on healthy subjects have supported CCM safety and efficacy; however, some negative side effects have been reported. In a dose-response study, seven subjects who received doses ranging from 500 to 12,000 mg and were monitored for 72 hours experienced diarrhea, headache, rash, and yellow stool.^36^ In another study, some subjects who received daily doses of 0.45 to 3.6 grams CCM for one to four months reported experiencing nausea and diarrhea.^37^ In the present study, oral administration of 100 mg/kg CCM for 30 days in rats caused no significant side effects throughout the experimental period. Based on our findings and the established CCM properties, the compound has a potential role as an adjunctive therapy to facilitate soft tissue repair in diabetic individuals.

## Conclusions

Our results show that CCM reduced acute inflammation and enhanced KGF expression in tooth extraction sockets under diabetic conditions. While our study did not reveal a significant impact of CCM on alveolar bone healing, further clinical research is needed to comprehensively evaluate its potential in this regard.

## Data Availability

All data generated or analyzed during this study are included in this published article
